# Interplay of endocrine and psychological factors in IVF/ICSI outcomes: a prospective cohort analysis

**DOI:** 10.3389/fendo.2025.1596664

**Published:** 2025-10-24

**Authors:** Chao Wang, Qian-Ling Li, Yun-Shuai Xu, Ke-Xin Cao, Ya-Qi Zhang, Liang Chang, Yue Tong, Ai-Jun Yang, Zhuang Liu, Lin Zhang, Li Lin, Tour Liu

**Affiliations:** ^1^ Division of Gynecology and Obstetrics, Peking University International Hospital, Beijing, China; ^2^ Department of Reproductive Medicine, Affiliated Hospital of Jining Medical University, Jining, China; ^3^ Department of Psychiatry, Shandong Daizhuang Hospital, Jining, Shandong, China; ^4^ Beijing Key Laboratory of Mental Disorders, National Clinical Research Center for Mental Disorders & National Center for Mental Disorders, Beijing Anding Hospital, Capital Medical University, Beijing, China; ^5^ Advanced Innovation Center for Human Brain Protection, Capital Medical University, Beijing, China; ^6^ Henan Key Laboratory of Medical Tissue Regeneration, School of Basic Medical Sciences, Xinxiang Medical University, Xinxiang, China; ^7^ School of Psychology and Mental Health, North China University of Science and Technology, Tangshan, Hebei, China; ^8^ Faculty of Psychology, Tianjin Normal University, Tianjin, China

**Keywords:** infertile women, psychological factor, stress, assisted reproductive technology (ART), reproductive outcomes

## Abstract

Infertility poses considerable challenges to both the endocrine system and psychological well-being of affected women. In particular, those undergoing Assisted Reproductive Technology (ART) confront not only physiological demands but also significant psychological stress. Despite the potential interplay between endocrine factors and psychological status in ART outcomes, large-scale investigations remain limited. In this prospective study, we enrolled 493 women undergoing *in vitro* fertilization and embryo transfer (IVF-ET) or intracytoplasmic sperm injection (ICSI). We collected baseline demographic data, reproductive history, treatment parameters, sleep quality, and psychological status (via validated questionnaires). We then evaluated the relationships between these variables and clinical pregnancy outcomes. Our analysis revealed that perceived stress, as measured by the Perceived Stress Scale (PSS), was a significant predictor of pregnancy outcomes, whereby elevated perceived stress correlated with lower pregnancy rates. In addition, endocrine and clinical parameters—specifically, basal serum LH levels, endometrial thickness on the day of embryo transfer, number of transferred embryos, and the proportion of top-quality embryos—were strongly associated with clinical pregnancy outcomes. These findings underscore the critical interplay between endocrinology and psychology in ART treatment. Elevated perceived stress and specific endocrine factors each exerted a notable impact on IVF/ICSI success, emphasizing the need for integrative approaches that address both physiological and psychological aspects to enhance clinical pregnancy rates.

## Introduction

Infertility is categorized as a disease by the World Health Organization and is defined as failure to achieve pregnancy within twelve months of unprotected intercourse or therapeutic donor insemination in women younger than 35 years or within six months in women who are older than 35 years (1, 2). In the last decades, the incidence of infertility is increasing. The trend of delaying marriage and childbearing has further exacerbated the burden of infertility. The prevalence of infertility ranges from 3.5 to 16.7% in high-resourced countries, and 6.9 to 9.3% in low-resourced countries. In the US, approximately 12.7% of reproductive age women seek treatment for infertility each year (2). And once recent research in China shows that the prevalence of infertility was 25% among couples of reproductive age, and more than half of the couples experiencing infertility would seek medical help (3).

Fertility treatments are complex, a significant number of couples resort to the Assisted Reproductive Technology (ART) (2). The use of ART has substantially improved the ability of couples with infertility to have biological children. ART has evolved rapidly since 1976, it includes intrauterine insemination (IUI),*In Vitro* fertilization and Embryo Transfer (IVF-ET), Intracytoplasmic Sperm Injection (ICSI), frozen Embryo Transfer (FET), Preimplantation genetic diagnosis (PGD)and etc. (4). Among ART, IVF-ET and ICSI is most widely used. In recent years, although many efforts have been made to improve the protocol of controlled ovarian hyperstimulation, optimize the laboratory culture system, and apply advanced molecular biology techniques, the current clinical pregnancy rate of IVF-ET is not satisfactory (5). In the year 2013, a survey involving 2,639 clinics in 75 participating countries with a global participation rate of 73.6% reported and estimated that 1,160,474 embryo transfers (ETs) were performed resulting in >344,317 babies (6). The IVF/ICSI combined delivery rates per fresh aspiration and frozen ET cycles were 24.2% and 22.8%, respectively. The cumulative delivery rate per aspiration was only 30.4% (6). That means that nearly 70% of the population have failed. Scientists and clinicians continue to explore the factors affecting clinical pregnancy, to achieve a high clinical pregnancy rate has been the common goal of doctors and patients.

Infertility, affecting millions globally, has significant physical, economic, and psychological implications on patients undergoing ART, including IVF. IVF is a multi-step procedure and will take much time and energy, and many factors can affect IVF failure and success rates (5). Previous studies on factors related to pregnancy outcome mainly focused on individual and clinical characteristics, for example, women's age and body mass index (BMI), genetics, serum levels of some hormones, sperm and egg characteristics, environmental and occupational factors and etc. (5, 7). Among them, the relationship between the psychological factor and infertility has been debated for years. There have been dozens of studies which have investigated the relationship between psychological symptoms prior to and during ART cycles and subsequent pregnancy rates, with conflicting results (8). While certain studies suggest no direct association between psychological symptoms and IVF clinical pregnancy rates, others emphasize the need for psychological evaluation and interventions for infertile couples, underlining the multifaceted nature of IVF treatments and outcomes. Moreover, Sleep disorders are prevalent in patients undergoing ART treatment (9–11) Although the sleep quality of females undergoing IVF/ICSI treatment is gaining more interest, sleep status prior to treatment is rarely assessed. The sample sizes of previous studies that investigated the incidence of sleep disorders before IVF/ICSI treatment were relatively small (11, 12). The relationship between the clinical features, the sleep quality and other psychosocial factors and the pregnancy outcome is limited. Our previous research has revealed that the sleep quality of infertile women before ovulation induction was poorer than that of normal adults (13). However, until now, the determinants that affect clinical pregnancy have not been fully revealed.

This study attempts to explore the effects of demographic characteristics, clinical characteristics, mental state and sleep conditions on the clinical pregnancy outcome of IVF-ET/ICSI, aim to provide clinical interventions before IVF/ICSI cycle for improving the clinical pregnancy rate.

## Materials and methods

### Participants and study design

This is a prospective cohort study. From April 2020 to July 2022, we recruited 493 females who planned to undergo IVF/ICSI treatment at the Reproductive Medicine Department, Affiliated Hospital of Jining Medical College, China. Inclusion criteria: a. Participants who had regular sexual intercourse without contraception for at least one year without pregnancy; b. Participants who planned to undergo IVF/ICSI; c. Age ≥20 years; d. Participants must be able to complete the questionnaire independently; e. No other serious physical illness. Exclusion criteria: a. History of major life events in the last 2 months; b. History of major mental illness. Each participant gave written informed consent, and all protocols were approved by the Medical Science Ethics Board of Affiliated Hospital of Jining Medical University (2020C081).

### Data collection

The baseline questionnaire requested details regarding general demographic and socioeconomic status, physical measurements, lifestyle habits, such as birth date, height, weight, educational level, income, smoking, alcohol use, drinking tea or cola, marriage and bearing status (e.g. length of marriage and cohabitation, pregnancy history including information concerning live births, miscarriage, induced abortions and stillbirths), seeking of medical help(e.g. number of previous IVF/ICSI cycles). Validated standardized scales were used to assess participants' psychological states including depression, anxiety, perceived stress, and sleep quality. The clinical data of IVF/ICSI treatment were extracted from the hospital information system, which included basal serum hormonal level, ovarian stimulation protocol, endometrial thickness on the day of embryo transfer, the number of oocytes retrieved, oocyte retrieval rate, number of mature oocytes, number of top-quality embryos, fertilization rate, and clinical pregnancy. The primary endpoint was clinical pregnancy, characterized by the cessation of menstruation, elevated levels of serum human chorionic gonadotropin (HCG), and the ultrasonographic visualization of a gestational sac with embryocardia-beats.

### Measures

One of the main challenges in assessing the mental state and sleep conditions in women with infertility is the accuracy of self-report measures. Face-to-face interviews were conducted by trained interviewers comprising psychiatrists and nurses. In the process of investigation, women were interviewed to recall information about their partners and themselves in private to assure the confidentiality of the information obtained.

#### Basic personal information questionnaire

The custom questionnaire collects demographic information such as age, body mass index, education level, economic status, lifestyle and fertility information such as duration of infertility, cause of infertility, infertility type, past pregnancy history and previous treatment.

#### Pittsburgh sleep quality index

Pittsburgh Sleep Quality Index (PSQI) was developed in 1989 by Buysse and colleagues and has been widely used to assess sleep quality (14, 15). PSQI consists of 19 items rated by self and 5 items rated by roommate or bedpartner. The 5 other-rated items were used for clinical information only and were not included in the scoring of PSQI. The 19 self-rated items are classified into seven components: subjective sleep quality, subjective sleep quality, sleep latency, sleep duration, habitual sleep efficiency, sleep disturbances, use of sleeping medication, and daytime dysfunction. Each component scores 0–3 and the scores of the seven components are added together to obtain a total PSQI score to measure global sleep quality. The Chinese version of PSQI showed great reliability and validity in previous study (16).

#### Beck depression inventory-short form

Beck Depression Inventory-Short Form (BDI-13) is widely used to evaluate the severity of depression. BDI consists of 13 items, and each item scores 0–3. The scoring assessment is as follows: 0–4 no depression, 5–7 mild depression, 8–15 moderate depression, 16–39 severe depression (17).

#### Self-rating anxiety scale

Zung's self-rating anxiety scale (SAS) assesses subjects' subjective feelings of anxiety (18). The scale includes 20 items that cover 4 groups of manifestations: cognitive, autonomic, motor, and central nervous system symptoms. Each item is rated on a Likert-type scale of 1–4, and the final score was equal to the integer part of the total score of 20 items multiplied by 1.25. Anxiety standard scores ≥ 50 were considered to be at risk for clinical anxiety (19).

#### Perceived stress scale

Perceived Stress Scale, which had demonstrated good reliability and validity (20), was developed in 1983 by American psychologist Cohent and contains two dimensions: sense of loss of control and tension. Each item is scored on a 5-point scale and the total score ranges from 0 to 56, with higher scores indicating greater perceived stress. The Cronbach's coefficient of the scale is 0.84–0.86, indicating good reliability (21, 22).

### Statistical analysis

Normally distributed continuous variables were expressed as mean ± standard deviation and categorical data were expressed as frequency and proportion. To assess the influences of demographic and reproductive characteristics, as well as IVF treatment characteristics on the clinical pregnancy, χ2, multiple logistic regression or Wilcoxon rank sum test were used for categorical variables. For the number of oocytes retrieved, the number of mature oocytes, and the number of high-quality embryos, a negative binomial distribution and log link function were specified. For oocyte retrieval rate and fertilization rate, a linear function was specified. To explore the potential effects of psychological characteristics and sleep status on clinical pregnancy, we used a logistic regression which include depression, anxiety, perceived stress, and sleep quality as independent variables (continuous). Our results reported odds ratios (OR) and 95% CI. OR < 1 indicates a reduced chance of the target event occurrence. Data were organized using Excel 2016 and analyzed using SPSS 27.0. The significance level was set at a two-tailed 5%.

## Results

### Demographics and fertility characteristics

This investigation encompassed 493 participants, delineating 272 women who achieved clinical pregnancy and 221 who did not. The median ages for male and female partners were established at 32 (range 29-37) and 32 (range 30-36), respectively. Among the cohort, 299 individuals (60.6%) had attained an education level of high school or above, 479 participants (97.2%) were identified as non-smokers or infrequent smokers, and 450 (91.3%) were non-drinkers or infrequent drinkers. The study identified 167 cases (33.9%) of primary infertility. A majority, 442 patients (89.7%), never had sought IVF/ICSI treatment. Two or more embryos transferred in 389 patients (78.9%). Median follicle-stimulating hormone (FSH) levels were recorded at 7.78 (range 6.67-9.19), luteinizing hormone (LH) at 4.77 (range 3.46-6.41), and endometrial thickness on the day of embryo transfer at 11 mm (range 9.2-12.5). Median scores for the Beck Depression Inventory (BDI), Self-Rating Anxiety Scale (SAS), Perceived Stress Scale (PSS), and Pittsburgh Sleep Quality Index (PSQI) were 1 (range 0-5), 28 (range 25-32), 20 (range 15-26), and 4 (range 2-5.5), respectively.

### Factor associated with IVF outcome

Demographic characteristic analysis (see **Table 1**) revealed no significant disparities between pregnant and non-pregnant women concerning BMI (*p* = 0.994), education level (*p* = 0.305), average monthly income (*p* = 0.632), smoking habits (*p* = 0.618), and alcohol consumption (*p* = 0.290). Conversely, differences in female and partner ages were statistically significant (*p* = 0.002 and *p* < 0.001, respectively), with habits of tea consumption showing marginal significance (*p* < 0.10). Given the important role of age, subgroup analyses were conducted to examine the predictive effects of age groups for both females and their male partners. Participants were divided into three age groups: Group 1 (≤30 years), Group 2 (>30 and ≤35 years), and Group 3 (>35 years). The logistic regression results indicated that differences in IVF outcomes across female age groups were not statistically significant (Group 1 vs 3: *β* = -0.09, *p* = 0.81, OR = 0.92; Group 2 vs 3: *β* = -0.09, *p* = 0.76, OR = 0.91). Conversely, male age was significantly associated with pregnancy outcomes. Compared with males in Group 3, those in Group 1 and Group 2 were less likely to experience unsuccessful IVF outcomes (Group 1 vs 3: *β* = -0.71, *p* = 0.04, OR = 0.49; Group 2 vs 3: *β* = -0.66, *p* = 0.03, OR = 0.52).

**Table 1:** Demographic characteristics between pregnant and not pregnant women.

Variable	Pregnant, n=272	Not pregnant, n=221	*P*-Value
Female Age(y), Median (IQR)	32 (29, 35)	33 (30, 37)	0.002
Male age(y),Median (IQR)	32 (29, 35)	33 (30, 37)	0.001
BMI (kg / m2 )	22.91(21.17, 25.73)	23.39 (20.81, 26.35)	0.994
Education, n(%)	272 (55.2)	221 (44.8)	0.305
Junior high School or below	101 (37.1)	93 (42.1)	
High school and above	171 (62.9)	128 (57.9)	
Income (yuan, per month), n(%)	272 (55.2)	221 (44.8)	0.632
<1000	10 (3.7)	11 (5.0)	
1000-2999	72 (26.5)	67 (30.3)	
3000-4999	116 (42.6)	80 (36.2)	
5000-9999	50 (18.4)	41 (18.6)	
>10000	24 (8.8)	22 (10.0)	
Smoking, n(%)	272 (55.2)	221 (44.8)	0.618
Never or rarely	266 (97.8)	213 (96.4)	
Smoking before pregnancy, quit now	4 (1.5)	6 (2.7)	
Smoking now	2 (0.7)	2 (0.9)	
Alcohol use, n(%)	272 (55.2)	221 (44.8)	0.290
Never or rarely	252 (92.6)	198 (89.5)	
Drinking before pregnancy, quit now	20 (7.4)	22 (10.0)	
Drinking now	0	1 (0.5)	
Drinking tea, n(%)	272 (55.2)	221 (44.8)	0.083
No	219 (80.5)	163 (73.8)	
Yes	53 (19.5)	58 (26.2)	
Drinking cola, n(%)	272 (55.2)	221 (44.8)	0.104
No	214 (78.7)	187 (84.6)	
Yes	58 (21.3)	34 (15.4)	

### Reproductive characteristics between pregnant and not pregnant women

Analysis of reproductive characteristics indicated no significant differences in infertility duration (*p* = 0.353), cause of infertility (*p* = 0.669), or number of previous IVF/ICSI treatment cycles (*p* = 0.850) between the groups. A statistically significant variance was noted in infertility type (*p* = 0.030) between pregnant and non-pregnant women (**Table 2**).

**Table 2:** Reproductive characteristics between pregnant and not pregnant women.

Variable	Pregnant, n=272	Not pregnant, n =221	*P*-Value
Infertility duration (y), Median (IQR)	2 (1, 4)	2 (1, 4)	0.353
Cause of infertility,n(%)			
Male factor	60 (22.1)	41 (18.6)	0.669
Female factor	129 (47.4)	109 (49.3)	
Both	51 (18.8)	48 (21.7)	
Unexplained	32 (11.8)	23 (10.4)	
Infertility type,n(%)			
Primary infertility	104 (38.2)	63 (28.5)	0.030
Secondary infertility	168 (61.8)	158 (71.5)	
Number of previous IVF/ICSI cycles,n(%)			
None	245 (90.1)	197 (89.1)	0.850
One or more	27 (9.9)	24 (10.9)	

IVF, *in vitro* fertilization; FET, frozen-thawed embryo transfer; ICSI, intracytoplasmic sperm injection;Primary infertility, defined as inability to achieve a spontaneous clinical pregnancy(The role of conception type in the definition of primary and secondary infertility.);Secondary infertility: defined as the inability to achieve a spontaneous clinical pregnancy following a previous spontaneous pregnancy(The role of conception type in the definition of primary and secondary infertility.).

### IVF treatment characteristics between pregnant and not pregnant women

IVF treatment characteristic analysis showed no significant differences in cycle type (*p* = 0.553; **Table 3**), FSH (*p* = 0.971), prolactin (PRL) (*p* = 0.381), estradiol (E2) (*p* = 0.372), testosterone (T) (*p* = 0.766), progesterone (P) (*p* = 0.663), total dose of gonadotropin (Gn) (*p* = 0.493), and duration of Gn used (*p* = 0.186) between the groups. Statistically significant differences were observed in basal serum LH level (*p* = 0.039), endometrial thickness on the day of embryo transfer(*p* = 0.005), number of embryos transferred (*p* < 0.001), and top-quality embryos rate (*p* < 0.001).

**Table 3:** IVF treatment characteristics between pregnant and not pregnant women.

Variable	Pregnant, n=272	Not pregnant, n =221	*P*-Value
Cycle type, n(%)			0.553
Fresh cycle	227 (83.5)	179 (81.0)	
Freezing cycle	45 (16.5)	42 (19.0)	
Basal serum hormonal level, Median (IQR)			
FSH (mIU/mL)	7.8 (6.65, 9.18)	7.77 (6.71, 9.23)	0.971
LH (mIU/mL)	4.56 (3.34, 6.13)	5.11 (3.65, 6.77)	0.039
PRL (ng/ml)	13.37 (9.21, 18.62)	12.75 (9.64, 16.5)	0.381
E2 (pg/ml)	39.05(29.36, 52.03)	41.72 (30.46, 51.87)	0.372
T(ng/ml)	0.41 (0.29, 0.54)	0.41 (0.28, 0.54)	0.766
P(ng/ml)	0.49 (0.32, 0.78)	0.48 (0.31, 0.73)	0.663
Total doses of Gn, Mean ± SD	2277± 772.5	2224.5± 777.8	0.493
Duration of Gn used, Median (IQR)	12 (10, 13)	12 (10, 13)	0.186
Endometrial thickness on the day of embryo transfer(mm), Median (IQR)	11 (9.5, 13)	10.5 (9, 12)	0.005
Number of embryos transferred, n(%)			
One	41 (15.1)	63 (28.5)	< 0.001
Two or more	231 (84.9)	158 (71.5)	
Top-quality embryos rate, Median (IQR)	0.88 (0.69,1)	0.79 (0.5, 1)	< 0.001

IVF, *in vitro* fertilization; FET, frozen-thawed embryo transfer; ICSI, intracytoplasmic sperm injection; R-ICSI, rescue intracytoplasmic sperm injection; Top-quality embryos were defined as 7 to 8 equally sized blastomeres with less than 10% fragmentation on day 3(Alpha Scientists in Reproductive Medicine and ESHRE Special Interest Group of Embryology. Istanbul consensus workshop on embryo assessment: proceedings of an expert meeting)FSH, follicle stimulating hormone; LH, luteinizing hormone; PRL, prolactin; E2,estradiol; T, testosterone; P, progesterone; Gn, gonadotropin.

### Psychological characteristics and Sleep status between pregnant and not pregnant women

The result of logistic regression indicated that only PSS had a significant *β* coefficient with *p*-value of 0.026 (see **Table 4**). A lower p-value indicates stronger evidence against the null hypothesis that PSS’ *β* coefficient is zero. This suggests that the observed result in the current sample is a rare event under the null hypothesis, implying that the null hypothesis may be rejected. Therefore, PSS is a significant predictor of pregnancy in this study. Specifically, participants with higher levels of perceived stress were less likely to become pregnant. In contrast, the predictive effects of depression (BDI score, *p* = 0.928), anxiety (SAS score, *p* = 0.357), and sleep quality (PSQI score, *p* = 0.766) were not significant.

**Table 4:** Influence of psychological characteristics and sleep status on clinical pregnancy.

Variable	*β*	*SE*	*P*-Value	*OR* (95% CI)
BDI	-0.003	0.03	0.928	0.997 (0.940, 1.058)
SAS	0.020	0.02	0.357	1.020 (0.978, 1.064)
PSS	-0.030	0.01	0.026	0.971 (0.946, 0.996)
PSQI	0.017	0.06	0.766	1.017 (0.910,1.136)

BDI, Beck Depression Inventory; SAS, Self-rating Anxiety Scale; PSS, Perceived Stress Scales; PSQI, Pittsburgh Sleep Quality Index; *β*, Coefficient of regression; *SE*, Standard Error; *OR*, Odds Ratio; CI, confidence interval.

## Discussion

Since the landmark birth of the first IVF baby in the United Kingdom in 1978, millions upon millions of children have been born since that time who simply could not have been without the development of IVF (23). IVF technology has seen widespread adoption for treating infertility, experiencing rapid advancements. Nonetheless, the current clinical success rates for IVF/ICSI are relatively modest, prompting considerable research interest in strategies to enhance clinical pregnancy rates in the realm of reproductive medicine. Our prospective study identified female age, male partner age, infertility type, basal LH, endometrial thickness on the day of embryo transfer, number of embryos transferred, embryo quality, and perceived stress level as significant factors influencing IVF pregnancy success in this cohort. Psychological factors like depression, anxiety, and poor sleep quality did not show a significant link. Perceived stress was the only significant psychological predictor, negatively impacting pregnancy chances. This study's strength lies in its comprehensive approach, examining social, physiological, and psychological variables that might affect the outcome of IVF-assisted pregnancies, thereby providing a theoretical foundation for strategies aimed at enhancing clinical pregnancy rates.

In our cohort of 493 participants, the clinical pregnancy rate reached 55.2%, modestly exceeding the 49.1% live birth rate per embryo transfer under 35 years old reported in the CDC's 2022 US national ART data. Given that our study exclusively assessed clinical pregnancy outcomes—without tracking subsequent live births—this discrepancy is methodologically expected. It further underscores a key limitation of our research: the absence of longitudinal follow-up for birth outcomes.

Consistent with existing literature, our study corroborates that the ages of both partners are significantly associated with differing clinical pregnancy outcomes. The fecundity of women decreases gradually but significantly beginning approximately at age 32 years and decreases more rapidly after age 37 years. The age-related decline in fertility is accompanied by significant increases in the rates of disorders that impair fertility, such as leiomyomas, tubal disease, and endometriosis, and an increased risk of aneuploidy and spontaneous abortion (24). However, the predictive effect of male age appears to be more pronounced in this study, whereas few studies have specifically examined the impact of paternal age on ART outcomes. Some studies have shown that advancing paternal age is associated with a lower probability of IVF/ICSI pregnancy (25), other studies have not observed a similar association (26–28). Recent investigations have elucidated the detrimental effects of paternal age on the timing of blastocyst formation and HCG levels, For every increase of one year, men had a 6% increased probability that the competent blastocyst was transferred on day 6 compared to day 5,and further they showed that the mean difference in hCG values when comparing paternal age group 30-34, 35–39 and 40–45 with the age group 25–29 in those receiving COS treatment, all showed significantly lower adjusted values for older men (29). These investigators demonstrate that after age 40 years sperm exhibit increased DNA fragmentation, chromatin decondensation and aneuploidy rates, and in IVF there are more canceled embryo transfers, lower clinical pregnancy rates and greater miscarriage rates (30).

The core findings of our investigation underscore the basal serum luteinizing hormone (LH) levels, endometrial thickness on the day of embryo transfer, the number of embryos transferred, and the proportion of top-quality embryos as pivotal factors associated with varied pregnancy outcomes. Intriguingly, an elevation in the basal serum LH levels has been identified as a prognosticator for the non-achievement of clinical pregnancy in women. Many studies have delved into the intricate role of hormone levels in determining the outcomes of IVF and intracytoplasmic sperm injection (ICSI) treatments. One area of focus has been that higher levels of basal LH, AMH, estradiol (E2) on gonadotropin-releasing hormone antagonist (GnRH-ant) start day and lower levels of LH on GnRH-ant start day had negative association with outcomes, including number of oocytes retrieved, 2PN, available embryos, incidence of OHSS, chemical pregnancy, clinical pregnancy, abortion and live birth; and the specific thresholds ratio of serum LH on the day of GnRH-ant administration to basal LH levels (hLH/bLH) may predict the success of IVF/ICSI outcomes more reliably (31). Another research found that the LH levels were correlated with clinical pregnancy and live births. The highest clinical pregnancy rate (25%) was achieved in women with low LH (< 2IU/l); whereas the miscarriage rate was almost similar in all the groups, indicating other factors that could influence the miscarriage rate exist. The pregnancy rate was the lowest (16%) in women with high LH levels (> 8IU/L); however, none of the results had statistical significance (32). Another study on serum LH measurements during the follicular phase in IVF cycles using GnRH agonist protocols indicates that suppressed levels of LH do not predict ovarian response or pregnancy outcomes (33). Consequently, the predictive effect of LH on pregnancy outcomes is still indeterminate in various studies. Furthermore, in our reach, the remaining basal serum hormonal level had no predictive value for pregnancy outcomes. This is consistent with previous studies. Basal levels of progesterone and testosterone have been scrutinized for their predictive value regarding ovarian response and IVF success. While elevated basal progesterone levels correlate with the incidence of preovulatory progesterone rise, they do not necessarily impact clinical pregnancy rates (34). Similarly, basal testosterone levels show a positive association with ovarian reserve markers and response, yet they fall short of predicting pregnancy outcomes (35, 36).

This investigation elucidated that augmented endometrial thickness at the time of transfer, the transfer of multiple embryos, and a heightened proportion of top-quality embryos show potential protective associations for the attainment of clinical pregnancy in females. The mean endometrial thickness observed on the day of embryo transfer was 11mm in the pregnancy cohort compared to 10.5mm in the non-pregnancy cohort. The relationship between endometrial thickness (EMT) and IVF outcomes, including live birth rates (LBR) and pregnancy losses, has been a focal point of numerous studies. The exact influence of EMT, as measured by ultrasonography, on IVF success rates remains a subject of debate due to inconsistencies across retrospective and prospective studies, confounded by factors such as age, estradiol levels, and oocyte count (37–39). Data from multiple sources, including the Canadian Assisted Reproductive Technology Registry and various fertility centers in China, indicate that increasing EMT is generally associated with improved clinical pregnancy rates and LBR, with a notable plateau effect beyond certain thickness thresholds (37, 40). What is he optimal EMT threshold that optimizes the IVF outcome? The results are controversial. Fresh embryo transfer cycles show an increase in LBR until an EMT of 10–12 mm, whereas in frozen embryo transfer (FET) cycles, the benefits plateau after 7–10 mm. Conversely, an EMT less than 6 mm is clearly linked with diminished LBR in both fresh and FET cycles (37). Another research reveals that clinical pregnancy rate, live birth rate and miscarriage rate may achieve their optimal level when EMT ≥ 12 mm, but some adverse pregnancy outcomes would be observed when EMT ≥15 mm especially for clinical pregnancy in fresh cycles (41). Despite these findings, the independent predictive value of EMT for IVF success is minimal, suggesting that while EMT is a factor, it should not be the sole criterion for clinical decision-making in IVF treatments (42).

Moreover, our investigation revealed strong associations between both embryo quality and the number of embryos transferred with clinical pregnancy outcomes, aligning with previous research findings. Similar studies have identified a non-linear relationship between the number of top-quality blastocysts (TQB) and successful pregnancy outcomes. Specifically, the odds of achieving an ongoing pregnancy or live birth increased by approximately 11% or more for each additional TQB, up to five TQB. Beyond this point, the outcomes plateaued, suggesting a diminishing return on pregnancy success after the transfer of five blastocysts (43). The contention over whether the augmentation in the number of embryos transferred is associated with improved pregnancy rates continues to be a subject of discussion. Another similar research supports that in women ≤42 years, transferring a single euploid blastocyst resulted in pregnancy rates similar to those of transferring 2 untested blastocysts while dramatically reducing the risk of twins (44). More and more studies emphasize the importance of single blastocyst transfer to minimize the risk of multiple pregnancies and enhance live birth rates, regardless of patient age or blastocyst quality. The American Society for Reproductive Medicine and the Society for Assisted Reproductive Technology have updated guidelines to reduce multiple pregnancies in IVF cycles, advocating for elective single-embryo transfers, particularly in women under 38, and suggesting preimplantation genetic testing to lower the risk of multiples (45).

Our analysis revealed that fertility-related characteristics, including the duration of infertility, its causes, and the number of preceding IVF/ICSI treatment cycles exhibited no significant association with clinical pregnancy rates. However, the type of infertility (based on the history of previous pregnancy) displayed a statistically significant correlation. This observation is in harmony with the results of some previous investigations, where these factors were associated with clinical pregnancy in several large-scale prospective studies. There was a significant decrease in age-adjusted livebirth rates with increasing duration of infertility between 1 and 12 years. Women who had had any previous pregnancy had a significantly higher livebirth rate than women with no previous pregnancies. The livebirth rate per treatment cycle, per egg collection, or embryo transfer was highest in the first cycle and decreased significantly with increasing number of previous treatment cycles (5). Notwithstanding the substantial participant count in our study, the sample size may still fall short of the requisite magnitude for a meaningful prediction of clinical pregnancy outcomes.

Lifestyle attributes such as BMI, educational attainment, income levels, smoking habits, tea, cola consumption, and alcohol intake were found to bear no significant relation to the outcomes of clinical pregnancy. Findings from multiple studies indicate that elevated BMI, particularly obesity, in women has been associated with poorer ART outcomes. A new approach, MitoScore, BMI, and body fat percentage were significantly lower in pregnant women as compared to non-pregnant women, they suggest that MitoScore, BMI, and body fat percentage could act as critical parameters in determining the success of ART (46). Not only spontaneous fertility and pregnancy outcomes after IVF, live birth rates both in IVF and ICSI cycles could be influenced by male and/or female BMI (47). However, conventional assessments of embryo morphology and quality did not significantly differ across BMI categories (48). Interestingly, the source of the oocytes (donor or non-donor) didn't sway the overall interpretation, but obesity in conjunction with polycystic ovary syndrome had a detrimental (49). The overarching consensus is that while elevated BMI may impact ART outcomes, its role as a sole determinant remains nuanced and multifactorial, pointing to the necessity of further research, especially on the benefits of weight reduction before undergoing ART.

The relationship between lifestyle choices and fertility outcomes, particularly in the context of IVF treatments, is a topic of growing research interest. A significant focus across several studies is on the consumption of caffeine and its potential impact on fertility. One New Zealand-based cross-sectional study examined the lifestyle and dietary habits of 250 women preparing for fertility treatment, revealing that a substantial proportion of these women engaged in poor lifestyle choices, notably high rates of alcohol (50.8%) and caffeine consumption (86.8%) (50). Another study delved into the specific effects of caffeine on IVF outcomes, using serum and follicular fluid samples from 619 women. While it found no direct link between coffee or tea consumption and pregnancy rates, it did observe potential adverse effects of caffeine on reproductive processes. Specifically, as serum caffeine levels increased, the number of eggs retrieved decreased, with higher coffee intake being associated with more aborted pregnancies (51). In contrast, a study involving 2474 couples and 4716 IVF cycles concluded that neither female nor male caffeine consumption impacted IVF outcomes significantly, such as live birth rates, fertilization rates, or implantation rates. A separate investigation into 2817 fertile women showed no discernible adverse effect of caffeine on time to conceive, further emphasizing the complexity of the caffeine-fertility relationship (52). Beyond caffeine, other lifestyle factors like smoking have also been scrutinized for their potential effects on fertility. One study pointedly demonstrated the negative impact of active smoking in women on both ovarian reserve and IVF outcomes. Active smokers had a decreased ovarian response to hyperstimulation, reflected in fewer mature oocytes retrieved, and a considerably lower clinical pregnancy rate when compared to non-smokers (53). However, neither caffeine nor smoking showed a significant association with pregnancy rates in the current study. One potential explanation is that unbalanced sample among subgroups related to smoking and drinking tea or cola. In conclusion, the connection between these lifestyles and successful fertility outcomes requires further research for definitive conclusions. The overarching sentiment, however, is the critical need for awareness and advice about healthy lifestyle choices for individuals planning to undergo fertility treatments.

IVF and ART are accompanied by significant emotional and psychological challenges. Our findings indicate that psychological states, especially perceived stress, might impact the rates of clinical pregnancy, being consistent with a meta-research’s result (54). It's noteworthy that perceived stress is not limited to the individual undergoing treatment. In IVF couples, research has shown that both partners may suffer more stress and emotional problems. This turbulence may even have biochemical indicators, as elevated cytokine levels in both partners have been associated with unsuccessful IVF attempts (55). There is also some indirect evidence to support this finding that the resilience of pregnant women in the IVF circle was significantly higher than that of non-pregnant women (56). Interestingly, this buffering effect seems to wane after unsuccessful IVF attempts (57). It could be inferred that a higher level of resilience may improve the success rate of IVF by buffering the increasing perceived stress, though with an unsustainable effect. Moreover, previous studies found the controversial influence of sleep quality and emotional problems on the outcome of IVF (58–60). The current study suggested that sleep quality and emotional problems may not be determining factors of IVF’s success. Specifically, the predictive effects of depression, anxiety, as well as sleep quality, which are regarded as stress-related health problems (61), were not significant. Compared with given emotional disorders or sleep disorders, subjectively perceived stress has a stronger connection to IVF’s outcome. Further research could explore whether resilience plays a buffering role in this connection.

## Limitations

This study has several limitations. Most notably, its single-center design may restrict the generalizability of the findings, while the relatively restricted sample size could further compromise the universality of our conclusions. This necessitates subsequent multi-center studies with expanded cohorts for validation. Additionally, although this study investigated the relationship between psychological factors, sleep quality, and pregnancy outcomes, the assessment was confined to the pre-IVF/ICSI treatment phase. Future research will incorporate dynamic evaluations at critical junctures throughout the assisted reproductive continuum—specifically during pre-oocyte retrieval, pre-embryo transfer, and peri-pregnancy confirmation periods. Through expanded sample sizes and enhanced data collection, we seek to comprehensively characterize the evolving trajectories of psychological states and sleep patterns during assisted reproduction. This integrated approach endeavors to establish an evidence base for clinical practice, providing targeted psychological interventions when indicated, with the ultimate objective of optimizing clinical pregnancy outcomes.

## Conclusion

There are several significant factors associated with the likelihood of achieving clinical pregnancy following IVF/ICSI treatment. Clinical pregnancy success was significantly associated with younger age in both partners, secondary infertility, lower basal LH levels, increased endometrial thickness at embryo transfer, transfer of more embryos, higher proportion of top-quality embryos, and reduced perceived stress. No significant associations were observed with lifestyle factors (BMI, smoking, alcohol), other hormonal profiles, infertility history, or psychological states except perceived stress.

## Data Availability

The original contributions presented in the study are included in the article/supplementary material. Further inquiries can be directed to the corresponding authors.
